# Macrophage-derived extracellular vesicles from *Ascaris lumbricoides* antigen exposure enhance *Mycobacterium tuberculosis* growth control, reduce IL-1β, and contain miR-342-5p, miR-516b-5p, and miR-570-3p that regulate PI3K/AKT and MAPK signaling pathways

**DOI:** 10.3389/fimmu.2024.1454881

**Published:** 2024-11-06

**Authors:** Giggil Pushpamithran, Robert Blomgran

**Affiliations:** Division of Inflammation and Infection, Department of Biomedical and Clinical Sciences, Faculty of Medicine and Health Sciences, Linköping University, Linköping, Sweden

**Keywords:** macrophage extracellular vesicles, miRNA, CREB1, MAPK13, SMAD4, tuberculosis, helminth-coinfection, inflammation

## Abstract

**Background:**

Helminth coinfection with tuberculosis (TB) can alter the phenotype and function of macrophages, which are the major host cells responsible for controlling *Mycobacterium tuberculosis* (Mtb). However, it is not known whether helminth infection stimulates the release of host-derived extracellular vesicles (EVs) to induce or maintain their regulatory network that suppresses TB immunity. We previously showed that pre-exposure of human monocyte-derived macrophages (hMDMs) with *Ascaris lumbricoides* protein antigens (ASC) results in reduced Mtb infection-driven proinflammation and gained bacterial control. This effect was entirely dependent on the presence of soluble components in the conditioned medium from helminth antigen-pre-exposed macrophages.

**Methods:**

Our objective was to investigate the role of EVs released from helminth antigen-exposed hMDMs on Mtb-induced proinflammation and its effect on Mtb growth in hMDMs. Conditioned medium from 48-h pre-exposure with ASC or *Schistosoma mansoni* antigen (SM) was used to isolate EVs by ultracentrifugation. EVs were characterized by immunoblotting, flow cytometry, nanoparticle tracking assay, transmission electron microscopy, and a total of 377 microRNA (miRNA) from EVs screened by TaqMan array. Luciferase-expressing Mtb H37Rv was used to evaluate the impact of isolated EVs on Mtb growth control in hMDMs.

**Results:**

EV characterization confirmed double-membraned EVs, with a mean size of 140 nm, expressing the classical exosome markers CD63, CD81, CD9, and flotillin-1. Specifically, EVs from the ASC conditioned medium increased the bacterial control in treatment-naïve hMDMs and attenuated Mtb-induced IL-1β at 5 days post-infection. Four miRNAs showed unique upregulation in response to ASC exposure in five donors. Pathway enrichment analysis showed that the MAPK and PI3K-AKT signaling pathways were regulated. Among the mRNA targets, relevant for regulating inflammatory responses and cellular stress pathways, CREB1 and MAPK13 were identified. In contrast, SM exposure showed significant regulation of the TGF-β signaling pathway with SMAD4 as a common target.

**Conclusion:**

Overall, our findings suggest that miRNAs in EVs released from helminth-exposed macrophages regulate important signaling pathways that influence macrophage control of Mtb and reduce inflammation. Understanding these interactions between helminth-induced EVs, miRNAs, and macrophage responses may inform novel therapeutic strategies for TB management.

## 1 Introduction

*Mycobacterium tuberculosis* (Mtb), primarily targeting macrophages in the lungs, remains a global health challenge where a quarter of all humans are believed to have a latent tuberculosis (TB) infection and over 10.6 million yearly develop active TB disease (1). More than two billion people are infected with intestinal helminths, and particularly soil transmitted helminths affect more than 1.5 billion people in Africa, Asia, and Latin America (2). It has been indicated that the pooled prevalence of helminth coinfection among TB cases is approximately 30% (3) with increased prevalence in high-burden settings (4). As helminths can modulate the immune response to Mtb, helminth/TB coinfection may alter TB progression and treatment efficacy, complicate TB diagnosis, and affect vaccine effectiveness (3, 5). Understanding the interaction between helminths and TB would aid in alleviating the public health challenges associated with the management of TB. During latent TB infection, the bacterium is no longer believed to “ lay” dormant, rather there is a constant immune battle to keep the bacteria in check where resident macrophages have an essential role in the containment of infection. Macrophages exhibit diverse functions and communication modalities, including the release of extracellular vesicles (EVs) containing biomolecules capable of modulating immune responses. Macrophages and dendritic cells are major producers of EVs (6). For instance, macrophage-released EVs during systemic candidiasis infection decrease the growth of *Candida* through the activation of ERK2 and p38 (7), and EVs from toll-like receptor (TLR) 3-activated macrophages confer anti-hepatitis C virus protection to hepatocytes (8).

In inflammatory diseases such as TB, EVs have emerged as important mediators recognized for regulating macrophage activation and polarization, where they have been shown to modulate the balance of M1/M2 macrophage polarization through alterations in glucose metabolism (9). The protective immune response against TB involves cell-mediated activation of macrophages by interferon-gamma-releasing CD4 T cells, as well as the efficient phagocytosis, autophagy, and other bactericidal traits of M1-polarized macrophages, which release proinflammatory cytokines such as TNF, IL-1β, and IL-6. During helminth/Mtb coinfection, there is an increase in regulatory networks that dampen Th1 responses and induce Th2-dominated immune responses, along with induction of regulatory T cells (10). Concurrently, there is an increased polarization of alternatively activated (M2) macrophages (11, 12), which are associated with tissue repair and anti-inflammatory functions that may have a reduced ability to control Mtb. However, it is not known whether helminth infection stimulates the release of host-derived EVs to induce or maintain their regulatory network that suppresses TB immunity.

By regulating gene expression, microRNAs (miRNAs) play a crucial role in modulating immune responses. miRNAs have been shown to influence innate immune responses, B-cell differentiation and antibody production, and T-cell development and function (13). For example, miR-146a, miR-21, and miR-155 have been described as principal regulators of inflammatory pathways in myeloid cells (14). Major host defense mechanisms against Mtb found regulated by miRNA include the triggering of apoptosis, induction of autophagy, and stimulation of interferon-gamma and TNF (15–17). Mtb can, however, manipulate these regulatory miRNA expression patterns to evade host immune responses, and so miRNAs have been implicated as biomarkers or regulators of immunity during TB (17).

Exploring EV-mediated miRNA transfer for immune regulation holds promise for novel TB therapeutic strategies. Coinfection with helminths and other neglected tropical diseases can influence the immune response, potentially impacting the efficacy of novel TB treatments utilizing miRNA or their targets (18). Therefore, it is crucial to assess how helminth exposure modulates miRNA expression in EVs. Previous studies have demonstrated helminth species-specific effects on TB immunity in endemic settings with controls and TB patients (19), as well as in human monocyte-derived macrophages (hMDMs) exposed to helminth antigens (11). Our recent research showed that hMDMs exposed to *Ascaris lumbricoides* antigens could mitigate Mtb infection-induced inflammation (e.g., release of IL-1β and IL-6) and enhance intracellular Mtb growth control when conditioned medium from antigen-pre-exposed cells was reintroduced post-Mtb infection (20). Therefore, this study aims to investigate whether EVs in conditioned medium from helminth antigen-exposed hMDMs mediate similar effects on treatment-naïve hMDMs and explore potential miRNAs that regulate the immune response to Mtb during helminth coinfection. Besides the *A. lumbricoides* protein antigen, we also used the *Schistosoma mansoni* soluble egg antigen for the generation of hMDM-conditioned medium and the exploration of the effect of EVs derived by helminth antigen exposure, as we previously observed that helminth species-dependent variations are induced in macrophages and other immune cells *in vitro* (11, 20) and in TB patients (10, 19, 21, 22).

## 2 Materials and methods

### 2.1 Ethics statement

Normal human serum (NHS) and buffy coat preparations from whole blood, the source of peripheral blood mononuclear cells (PBMCs) and monocytes, were obtained from healthy volunteers from Linköping University Hospital Blood Bank and Jönköping Hospital Blood Bank. All donor samples were de-identified and anonymized before being provided to the researchers, ensuring complete confidentiality. All the work was carried out in accordance with the Declaration of Helsinki, not requiring a specific ethical approval according to paragraph 4 of the Swedish law.

### 2.2 Helminth antigens

Whole worm protein extracts of *A. lumbricoides* (ASC) from Allergen AB Thermo Fisher Scientific and *S. mansoni* soluble egg antigen (SM) donated by Professor Mike Doenhoff, Nottingham University, Nottingham, UK, were used. The protein concentration of each antigen was determined by Bradford assay and stored at −80°C until used. Concentration and pre-exposure time of helminth antigen was set based on our previous study (20), and that of the work with macrophage exposure by others (23).

### 2.3 Generation of EV-free cell culture medium

EV-free cell culture medium was prepared by ultracentrifugation of NHS pooled from five donors at 120,000 × *g* overnight at 4°C consecutively two times. The supernatant from double ultracentrifuged NHS was then added at 10% to DMEM medium with 10 mM HEPES and 1% L-glutamine used to prepare the medium, which was followed by filtration with a 0.22-μm Stericup quick-release vacuum-driven filtration system (Millipore, Darmstadt Germany). This EV-free cell culture medium was used for all incubations of mature macrophages (hMDMs), from generating conditioned medium, preparation of bacteria and infection experiments, and resuspending isolated EVs for functional experiments.

### 2.4 Generation of hMDMs

hMDMs were generated following the methodology outlined in previous studies (20). In brief, PBMCs isolated from buffy coats were plated and allowed to adhere for 1.5–2 h. Non-adherent cells were removed with warm Krebs-Ringer Phosphate buffer with glucose, and adherent monocytes were allowed to differentiate for 6 days with fresh complete DMEM (containing 10% NHS pooled from five donors, HEPES, L-glutamine, penicillin, and streptomycin, without specific addition of growth factors) that was replenished on the third day of culture. At day 6, mature macrophages (hMDMs) were detached using trypsin and plated in triplicate at 100,000 cells per well in 96-well plates in antibiotic-free EV-free cell culture medium and hMDMs were rested overnight before infected with Mtb. For generating cell culture supernatants, which were the source of conditioned medium used for further isolation and characterization of hMDM-released EVs, day 7 hMDMs were treated with or without 5 µg/mL of helminth antigens in EV-free cell culture medium for 48 h.

### 2.5 Bacterial preparation, infection, and luciferase measurement

Bacterial preparation and infection of hMDMs were performed according to our previous study (20). In brief, we used Mtb H37Rv (ATCC) carrying a luciferase construct cultured in Middlebrook 7H9 broth supplemented with 0.5% Tween 80 and 10% albumin-dextrose-catalase enrichment (ADC; Becton Dickinson, Franklin Lakes, NJ, USA) and 100 µg/mL hygromycin (Sigma) at 37°C to log phase. For infection experiments, Mtb was prepared in EV-free cell culture medium and treatment-naïve hMDMs infected with a multiplicity of infection of four bacteria per cell for 1.5 h, before these hMDMs were washed and incubated for 5 days in EV-free cell culture medium with or without ultracentrifuge preparations of hMDM-derived EVs or conditioned medium depleted of EVs. Mtb luciferase was measured for quantifying Mtb replication, and the luciferase signal in both the supernatant and cell lysate were measured using decanal (Sigma-Aldrich, St. Louis, MO, USA) as the substrate. The luciferase signal from uninfected hMDMs was subtracted from the Mtb luciferase signal in infected samples to account for background noise. To determine the total Mtb growth in each well, the relative luminescence values from the lysate and supernatant were combined. The median value of each triplicate was then either expressed as the absolute value of relative luminescence units or normalized to the day 0 medians (day of infection) from the same donor and treatment, generating an Mtb-fold change relative to day 0.

### 2.6 Isolation of macrophage EVs

EVs were harvested from the cell culture supernatant of hMDMs stimulated with helminth antigen for 48 h. In brief, 48-h cell culture supernatants were centrifuged at 400 × *g* for 10 min at 4°C to clear cells and debris, named conditioned medium throughout. The conditioned medium was then centrifuged again at 2,000 × *g* for 20 min at 4°C to remove apoptotic vesicles, followed by ultracentrifugation with Beckman Coulter Optima L-80XP at 200,000 × *g* for 2 h at 4°C to isolate EVs. The pelleted EVs used in functional experiments were here resuspended in EV-free cell culture medium and added post-infection with Mtb. However, for characterization purposes, the pellets obtained after initial ultracentrifugation were additionally washed with EV-free PBS (DPBS filtered through a 0.22-μm pore size Stericup quick-release sterile vacuum-driven filtration system) at 200,000 × *g* for 2 h at 4°C. Subsequently, these pellets were resuspended in either EV-free PBS for characterization or QIAzol for isolation of total RNA/miRNA. For evaluating the functional role of EVs, a conditioned medium without EVs was simultaneously generated. In brief, supernatant collected from the initial ultracentrifugation was again centrifuged at 7,500 × *g* for 15 min at RT in 100-kDa Amicon Ultra concentrate filters to remove particles > 30 nm and used as EV-free conditioned medium.

### 2.7 Characterization of macrophage EVs with transmission electron microscopy

EV samples fixed with 1%–2% paraformaldehyde were analyzed using transmission electron microscopy at the Linkoping University Core Facility. EVs were identified and visualized using negative staining. In brief, 5 μl of samples was mounted to a hydrophilic formvar- carbon-coated, 300-mesh, copper electron microscopy grid (TED PELLA, Inc), and grids were washed, blotted, and negatively stained with 2% uranyl acetate. Electron micrographs were obtained using 80-kV transmission electron microscopy (JEOL JEM 1400 Flash, JEOL LTD, Tokyo, Japan).

### 2.8 EV characterization using the MACSPlex Exosome kit

Flow cytometric characterization of EVs was performed using the MACSPlex Exosome kit from Miltenyi Biotec (order no. 130-122-209), following the manufacturers instruction. In brief, each sample containing 6 µg of EVs was incubated with 15 µL of MACSPlex Exosome Capture Beads before being washed with MACSPlex buffer and subsequently incubated with 15 µL of MACSPlex Exosome Detection Reagent cocktail (consisting of anti-CD9, anti-CD63, and anti-CD81) in the dark at 4°C for 1 h. Then, samples were washed twice with MACSPlex buffer and acquired by a Gallios flow cytometer (Beckman Coulter), and data were analyzed using Kaluza 2.1 and presented after being normalized to the median signal intensity obtained from the buffer.

### 2.9 EV characterization with Western blot analysis

To confirm that the pellets from ultracentrifuged conditioned medium contained EVs and were free of cellular contamination, samples underwent Western blot analysis. EV samples were boiled for 5 min at 95°C with equal volume of 2× Laemmli sample buffer containing 5% 2-β-mercaptoethanol and freshly added dithiothretol (NuPAGE sample reducing agent). Proteins in EV lysates were separated by SDS-polyacrylamide gel electrophoresis and transferred onto nitrocellulose membranes (Merck). Nitrocellulose membranes were blocked with 5% dry milk in PBS containing 0.075% Tween 20 for 1 h at RT. Membranes were immunoblotted overnight at 4°C with 1:2,000 diluted rabbit anti-human calnexin (Abcam, ab92573) and mouse anti-human flotillin-1 (BD Sciences, cat. 610820). After washing with PBS containing 0.075% Tween 20, the membrane was incubated with Alexa Fluor 680 goat anti-rabbit and Alexa Fluor 790 goat anti-mouse secondary antibodies (Invitrogen, 1:1,000 diluted) for 1 h. After washing, protein bands were identified with the Odyssey LI-COR CLX imaging system.

### 2.10 NTA measurement with Nanosight NS300

EV samples used for the nanoparticle tracking analysis (NTA) were diluted in PBS to a final volume of 1 mL. Ideal measurement concentrations were found by pre-testing the ideal particle per frame value (20–100 particles/frame). The following settings were used according to the manufacturer’s software manual (NanoSight NS300 User Manual, MAN0541-01-EN-00, 2017): camera level increased until all particles were distinctly visible not exceeding a particle signal saturation. The ideal detection threshold was determined to include as many particles as possible with the restrictions that 10–100 red crosses were counted while only <10% were not associated with distinct particles. Blue cross count was limited to 5. Autofocus was adjusted so that indistinct particles were avoided. For each measurement, five 1-min videos were captured under the following conditions: cell temperature: 21°C; syringe speed: 30 µL/s. After capture, the videos were analyzed by the in-build NanoSight Software NTA 3.4 with a detection threshold of 4. Hardware: embedded laser: 45 mW at 488 nm; camera: sCMOS. The count of finalized tracks consistently exceeded the suggested lower limit of 1,000 to minimize data skewing based on single large particles (24).

### 2.11 miRNA isolation

For isolation of miRNA, EV pellets from ultracentrifugation were resuspended and homogenized in QIAzol lysis reagent and stored at −80°C until RNA isolation. Total RNA was isolated using the miRNeasy Micro kit (cat. no. 217084, Qiagen) according to the manufacturer’s protocol to include all miRNA. In brief, QIAzol-lysed samples were thawed slowly on ice and incubated at RT for 5 min. At this, time control oligos (cel-miR-39-3p and ath-miR1591) were added and mixed, followed by vigorous shaking with chloroform, and incubated for 3 min at RT. After 15 min of centrifugation, the upper aqueous phase was resuspended in 1.5 times the volume of 100% ethanol and transferred to RNeasy MinElute spin columns. Using RWT, RPE buffer, and 80% ethanol, spin columns were washed briefly under centrifugation. miRNA in the spin columns was eluted with 14 µL of RNase-free water by centrifugation at full speed for 1 min. The RNA concentration was determined with an Agilent 2100 Bioanalyzer using the RNA Pico chip.

### 2.12 Quantitative real-time PCR

miRNA expression profiling was conducted using the TaqMan Advanced miRNA Human A Card (A34714; Thermo Fisher Scientific) following the manufacturer’s instructions. The assay was performed using the QuantStudio 7 Flex Real-Time PCR system (Applied Biosystems). Data analysis was carried out using QuantStudio 3D Analysis Suite software version 3.1.6 (Life Technologies Corporation), a web-based tool that employs the comparative Cq (ΔΔCq) method for quantifying relative gene expression across samples. Relative quantification (RQ) or fold change (FC) was determined from the Cq values using the equation RQ = 2^−ΔΔCq^. Endogenous controls provided in the TaqMan Advanced miRNA assay card were utilized for data normalization. hsa-miR-16-5p, which is a stable miRNA in cell line, was used as endogenous control (25).

### 2.13 Bioinformatics analysis

miRNA (RQ > 2) targets were extracted from mirWalk (miRWalk.umm.uni-heidelberg.de), based on the following conditions. Targets were selected if present in two of the three data bases, e.g., if targets were commonly present in TargetScan and miRDB, or if commonly present in mirTarBase (validated miRNA targets) and miRDB. These targets were pooled and used for functional enrichment analysis and GO (gene ontology) analysis by utilizing the web-based gene set analysis toolkit (WebGestalt, www.webgestalt.org) (26).

### 2.14 Statistical analysis

All statistical analyses were performed with Graph Pad Prism 8.4.3 (686). The data were presented as mean ± SEM and analyzed using ANOVA and Student’s *t*-test. Graphs prepared using ggplot2 in R studio 2023.12.1 are indicated in figure legends.

## 3 Results

### 3.1 Characterization of macrophage-released EVs

**Figure 1:**
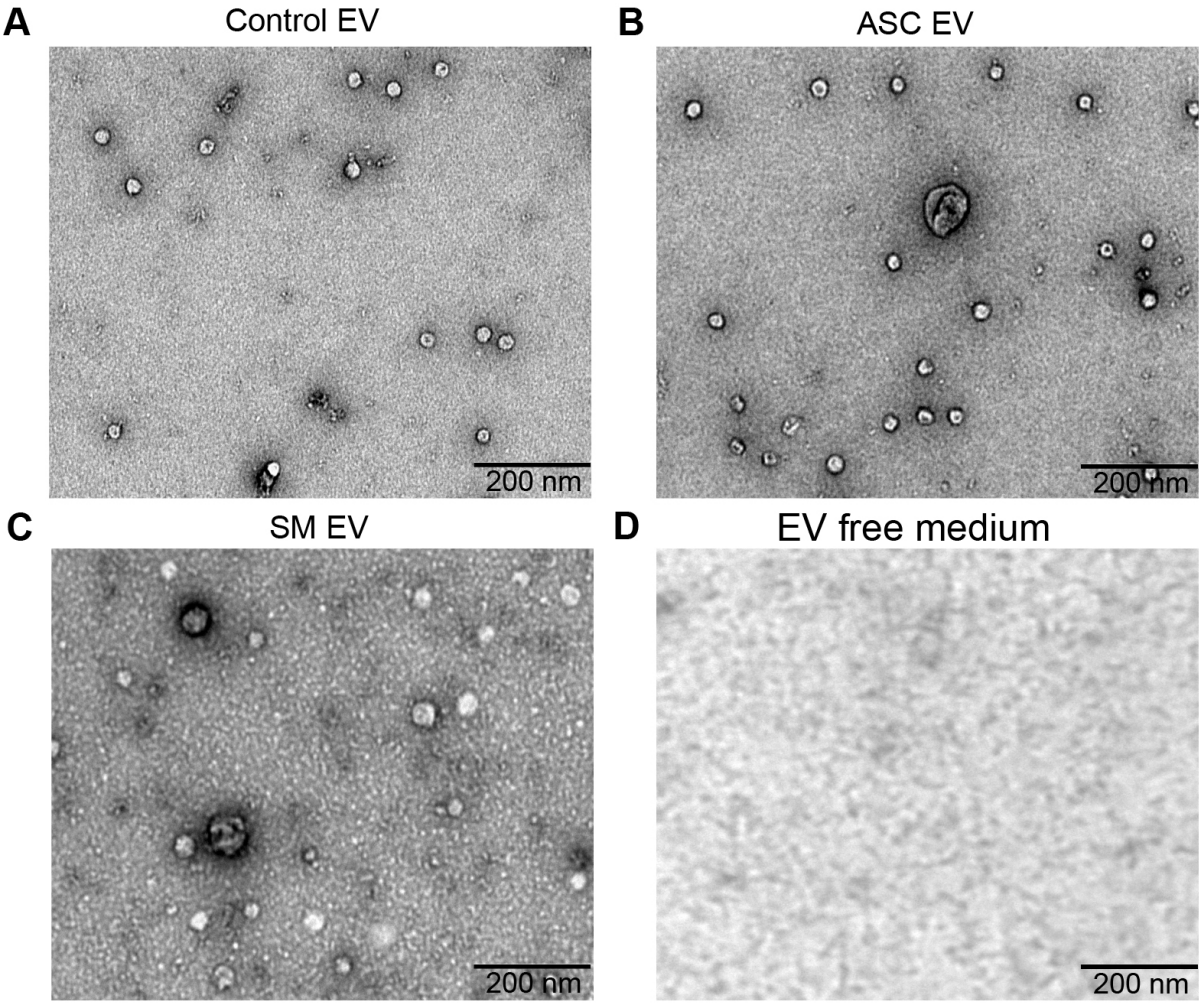
Confirmation of extracellular vesicles (EVs) released from human macrophages. Transmission electron microscopy (TEM) images of EVs released from healthy human monocyte-derived macrophages (hMDMs) that are unexposed **(A)**, 48 h 5 µg/mL *Ascaris lumbricoides* antigen exposed (ASC) **(B)**, 48 h 5 µg/mL *Schistosoma mansoni* antigen exposed (SM) **(C)**, or EV-depleted medium used for culturing of hMDMs **(D)**. Magnification 20,000×; the size of the scale bar is indicated in micrographs. Representative of *n* = 5 donors.

**Figure 2:**
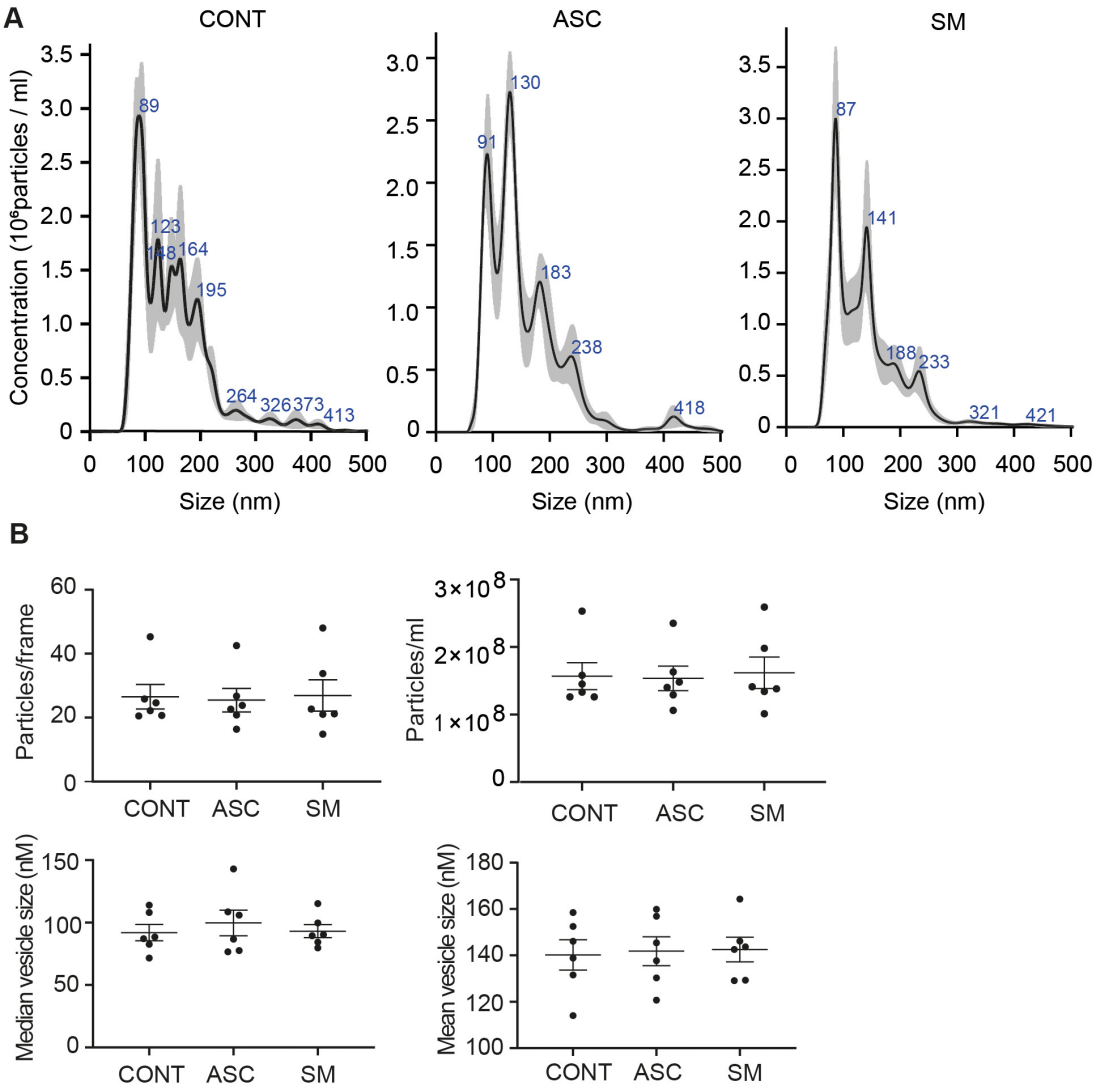
Characterization of hMDM-derived EVs using nano particle tracking assay (NTA). Five 1-min videos captured per sample, merged, and average reported. Representative NTA analysis **(A)**. Scatter plot graphs of concentration of EVs per frame, concentration of EVs per milliliter, median size, and mean size of released EVs **(B)**; no statistical difference was observed between the treatment using one-way ANOVA. Data expressed as means ± SEM from *n* = 6 independent donors.

**Figure 3:**
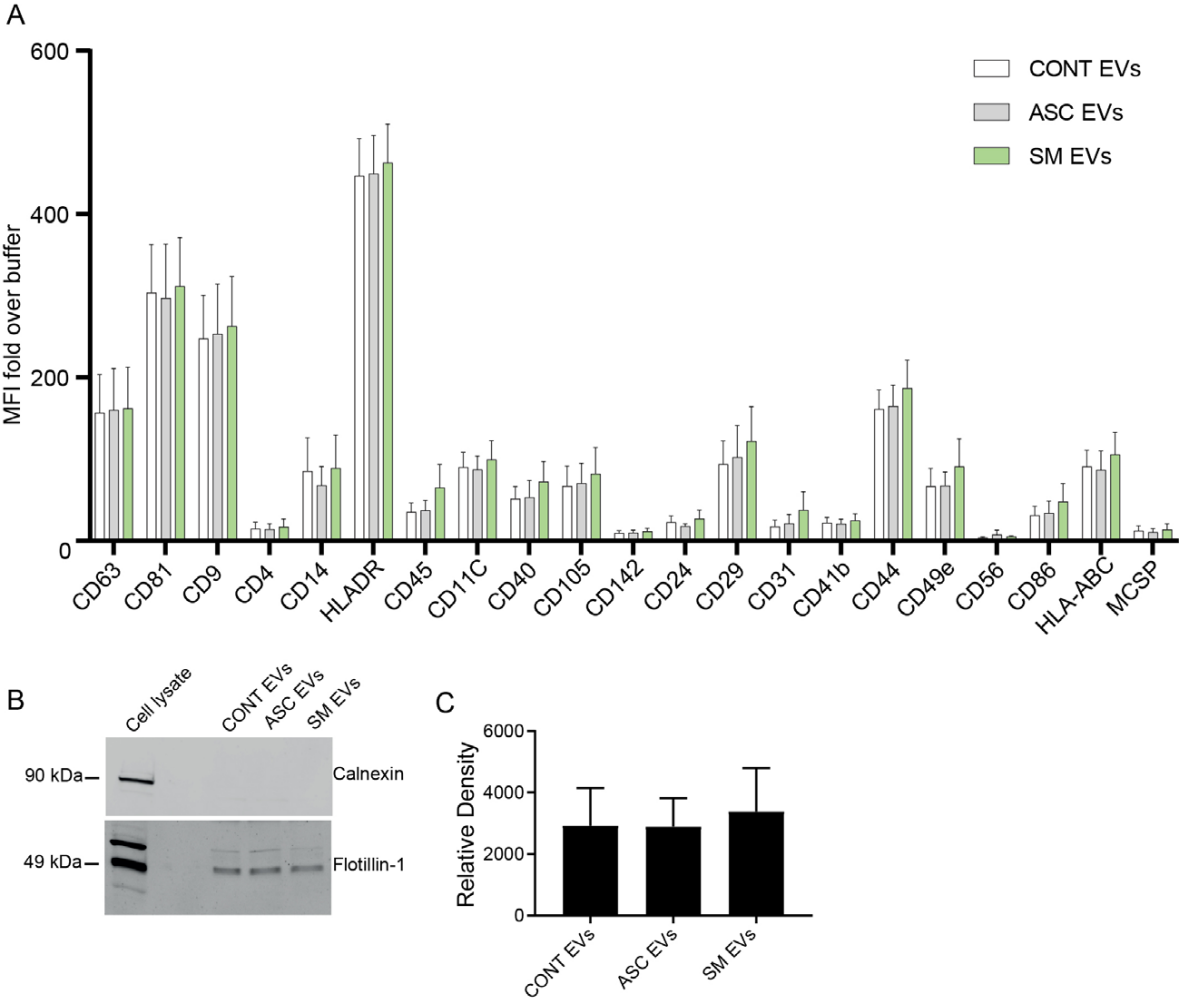
Characterization of EVs using the MACSPlex Exosome kit and Western blotting for flotillin-1. **(A)** Median fluorescence intensity (MFI) fold over buffer from different donors (*n* = 4) reported for markers twofold and above with the MACSPlex Exosome kit. No statistical difference was observed between the treatments using one-way ANOVA. Data expressed as means ± SEM. Isolated EVs carry flotillin-1 but not the impurity marker calnexin **(B, C)**. Isolated EVs were lysed in sample buffer and subjected to Western blot using anti-flotillin-1 (EV marker) and anti-calnexin (endoplasmic reticulum protein) **(B)**. Full scan of the entire original gel(s) (**Supplementary Figure S5**). Relative density of flotillin-1 **(C)**. Data expressed as means ± SEM from *n* = 8 independent donors.

Previously, we observed that cell-free culture supernatants from 48-h helminth antigen-exposed hMDMs can modulate intra-macrophage Mtb growth control and infection-driven proinflammation (20). In this study, we explored the role of EVs in mediating this effect. To generate the conditioned medium of helminth-exposed macrophages, similar to our previous work, hMDMs were exposed to *A. lumbricoides* protein antigen (ASC), *S. mansoni* soluble egg antigen (SM), or left unexposed (control) for 48 h. EVs isolated from the conditioned medium of macrophage culture supernatant using ultracentrifugation were verified through a combination of methodologies to ascertain the true identification of EVs, based on the MISEV2018 Guideline (27). Transmission electron microscopy was used for generating images of EVs at high resolution. The characteristic round double-membrane structures of EVs with different sizes were observed in all samples of macrophage-derived materials, and EV-free medium used for culturing of hMDMs showed no presence of vesicles (**Figure 1**). To confirm the biophysical features of EVs, further characterization of the EVs was performed using nanoparticle tracking assay (NTA) that utilizes light scattering properties for the determination of vesicle size. This demonstrated that EVs from all treatments exhibited an average size ranging from 50 to 150 nm (**Figure 2**), in line with them being EVs (27). The MACSPlex exosome kit in combination with flow cytometry analysis was additionally performed to evaluate the surface marker expression of EVs. This demonstrated the presence of canonical surface markers for EVs, including CD63, CD81, and CD9 on all isolated EVs (28) (**Figure 3A**). Notably, HLADR, a recognized indicator of antigen presentation and immune modulation, was highly expressed on the surface of macrophage-derived EVs. There was no difference in the marker expression on hMDMs EVs derived from the different treatments. To assess the purity of the isolated EVs, we further analyzed the presence of the typical EV marker flotillin-1 and the impurity marker calnexin by Western blotting (**Figures 3B, C**). Lysates from all isolated EVs expressed the specific EV marker flotillin-1, without having calnexin, indicating the high purity of isolated EVs.

### 3.2 Extracellular vesicles from helminth exposure enhance Mtb growth control in hMDMs

**Figure 4:**
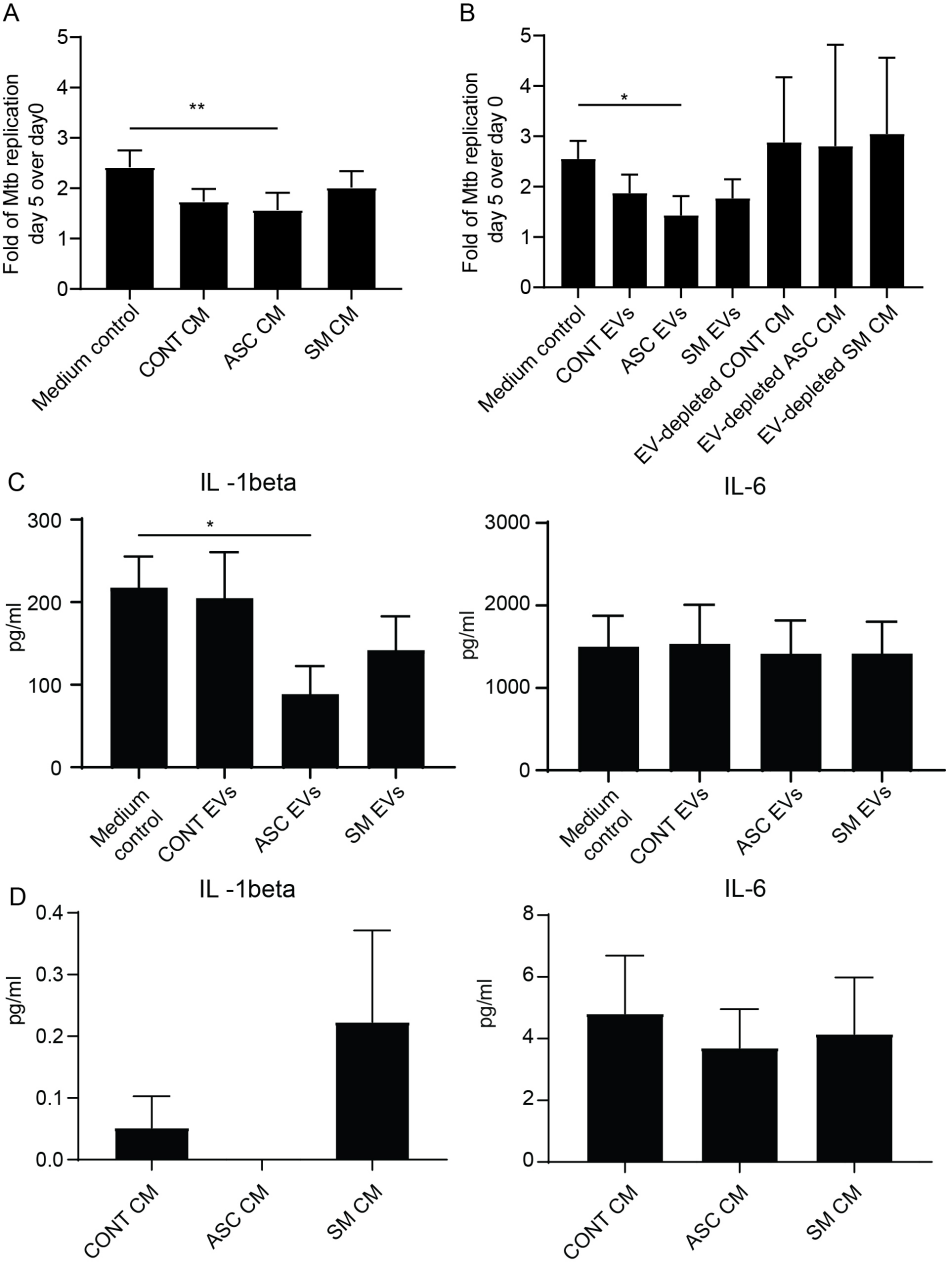
EVs from ASC-exposed hMDMs inhibit Mtb growth and lower infection-driven IL-1β. Treatment-naïve hMDMs were infected with luciferase-expressing H37Rv at a multiplicity of infection of four bacteria per cell for 1.5 h. Extracellular bacteria were removed and 50% of 48-h conditioned medium (CM) from different treatments was added, *n* = 6 **(A)**, or isolated extracellular vesicles from these CM, *n* = 6, were added **(B)**. Total luciferase values calculated by combining luminescence signal from supernatant and lysate are shown. Day 5 post-Mtb infection levels of IL-1β and IL-6 in cell-free culture supernatants of untreated hMDMs (Medium control) and extracellular vesicle (EV)-treated **(C)**, and pre-infection levels of IL-1β and IL-6 in the conditioned medium (CM) used to isolate EVs for B **(D)**. Data expressed as mean ± SEM from six independent donors with *p < 0.05 and **p < 0.01 using one-way ANOVA with Dunn’s multiple correction. CONT EVs, isolated EVs from 48-h conditioned medium of unexposed hMDMs; ASC EVs, isolated EVs from 48-h conditioned medium of ASC-treated hMDMs; SM EVs, isolated EVs from 48-h conditioned medium of SM-treated hMDMs.

In our previous study, we found that adding back the conditioned medium from 48-h helminth antigen pre-exposed macrophages to the same cells after Mtb infection resulted in a 50% reduction of the total bacterial load compared to untreated on day 5 post-infection (20). Utilizing a similar experimental setup, we explored the effect of conditioned medium and capacity of isolated helminth-induced macrophage EVs to modulate the growth of Mtb within macrophages that had not previously been exposed to helminth antigens, i.e., treatment naïve. The addition of the conditioned medium resulted in an increased Mtb growth control at day 5 post-infection, which was only significant with the ASC conditioned medium (**Figure 4A**). More importantly, bacterial growth control was also significantly increased when isolated EVs from the ASC conditioned medium (ASC EVs) were added to unexposed macrophages. To substantiate our finding, we simultaneously used a conditioned medium that was depleted of EVs, which showed a total loss in Mtb growth control (**Figure 4B**). This shows that EVs from helminth-exposed macrophages indeed can affect the intra-macrophage growth of virulent Mtb. Conditioned medium or isolated EVs from preparations of SM-exposed hMDMs did not affect bacterial growth to the same extent, indicating a helminth species-dependent capacity in modulating Mtb growth control in macrophages. Similar to our previous finding of reduced Mtb infection-driven proinflammation provided by conditioned medium from the ASC pre-exposed hMDMs (20), we observed a significant reduction in IL-1β at day 5 post-infection when macrophage EVs from ACS exposure were added to treatment-naïve hMDMs after Mtb infection (**Figure 4C**). Conditioned medium from which EVs were isolated contained no IL-1β (**Figure 4D**). This indicates that EVs from helminth-exposed macrophages can regulate inflammation in TB.

### 3.3 Identification of differentially expressed miRNA in EVs of helminth-exposed macrophage

The TaqMan advanced miRNA human A card that detects the 377 most common mature human miRNAs was used to analyze the miRNA in isolated EVs of helminth antigen-exposed macrophages obtained from five donors. Out of these miRNAs, 214 and 213 were detectable in EVs from ASC and SM exposure, respectively. A total of 75 miRNAs were upregulated with ASC compared to control, and 70 were upregulated with SM compared to the control. miRNAs were deemed upregulated if their RQ-fold change was ≥ 2. Several miRNAs were overlapping between the two helminth antigen exposures, and 39 miRNAs showed unique upregulation in response to ASC exposure, while 34 uniquely expressed miRNAs were identified following SM exposure in the pooled data for the donors. Subsequently, we explored the presence of miRNAs commonly upregulated in all donors after ASC or SM exposure. Our analysis revealed that miR-342-5p, miR-516b-5p, miR-570-3p, and miR-188-3p were commonly upregulated in EVs from ASC-exposed hMDMs across all donors. For SM exposure, miR-296-5p and miR-452-5p were commonly upregulated across all donors (**Table 1**). The heat map for the differential expression of all 377 miRNAs following helminth antigen exposure displayed a vast donor variation (**Supplementary Figure S1**).

**Table 1:** Commonly upregulated miRNA in EVs of helminth antigen-exposed hMDMs across all donors.

Commonly upregulated miRNA with ASC exposure			
miRNA	CONT RQ	ASC RQ	SM RQ
**hsa-miR-342-5p** **478044_mir**	1	139.3821	1.427668
**hsa-miR-516b-5p** **478979_mir**	1	12.75203	1.844326
**hsa-miR-570-3p** **479053_mir**	1	70.40454	0.329303
**hsa-miR-188-3p** **477942_mir**	1	9.70081	4.69554*
Commonly upregulated miRNA with SM exposure			
miRNA	CONT RQ	ASC RQ	SM RQ
**hsa-miR-296-5p** **477836_mir**	1	13.39489*	547.9478
**hsa-miR-452-5p** **478109_mir**	1	2.515781*	5.023398

ASC, miRNA in EVs from *Ascaris lumbricoides* antigen-exposed hMDMs; SM, miRNA in EVs from *Schistosoma mansoni* antigen-exposed hMDMs; RQ, RQ-fold. *RQ-fold below 2 in one or more donors.

### 3.4 miRNAs from EVs show distinct modulation of inflammatory pathways

**Figure 5:**
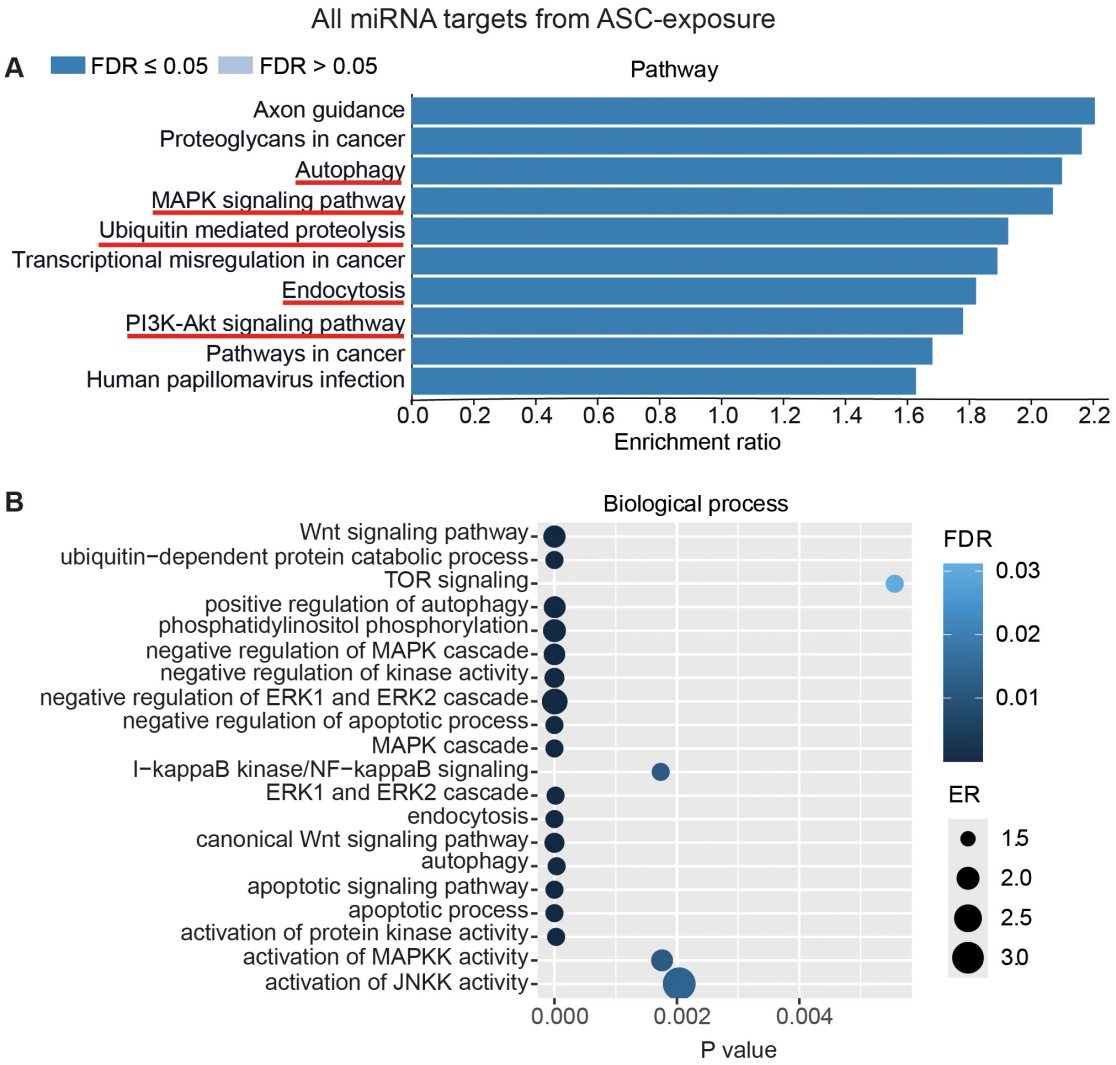
miRNAs target gene KEGG pathway enrichment analysis and GO analysis for ASC exposure. miRNAs with RQ-fold ≥ 2 from all donors were subjected to target prediction (miRWalk), and common targets across all donors were identified and used to detect pathways and biological processes that were affected. Top 10 enriched pathways using the KEGG pathway from Webgstalt **(A)**, and GO analysis of common target genes for 20 biological processes **(B)**. ER, enrichment ratio; FDR, false discovery rate. Underlined pathways in **(A)** were found modulated in all five donors when analyzing each donor separately. Graph in **(B)** was generated using ggplot2 in R.

**Figure 6:**
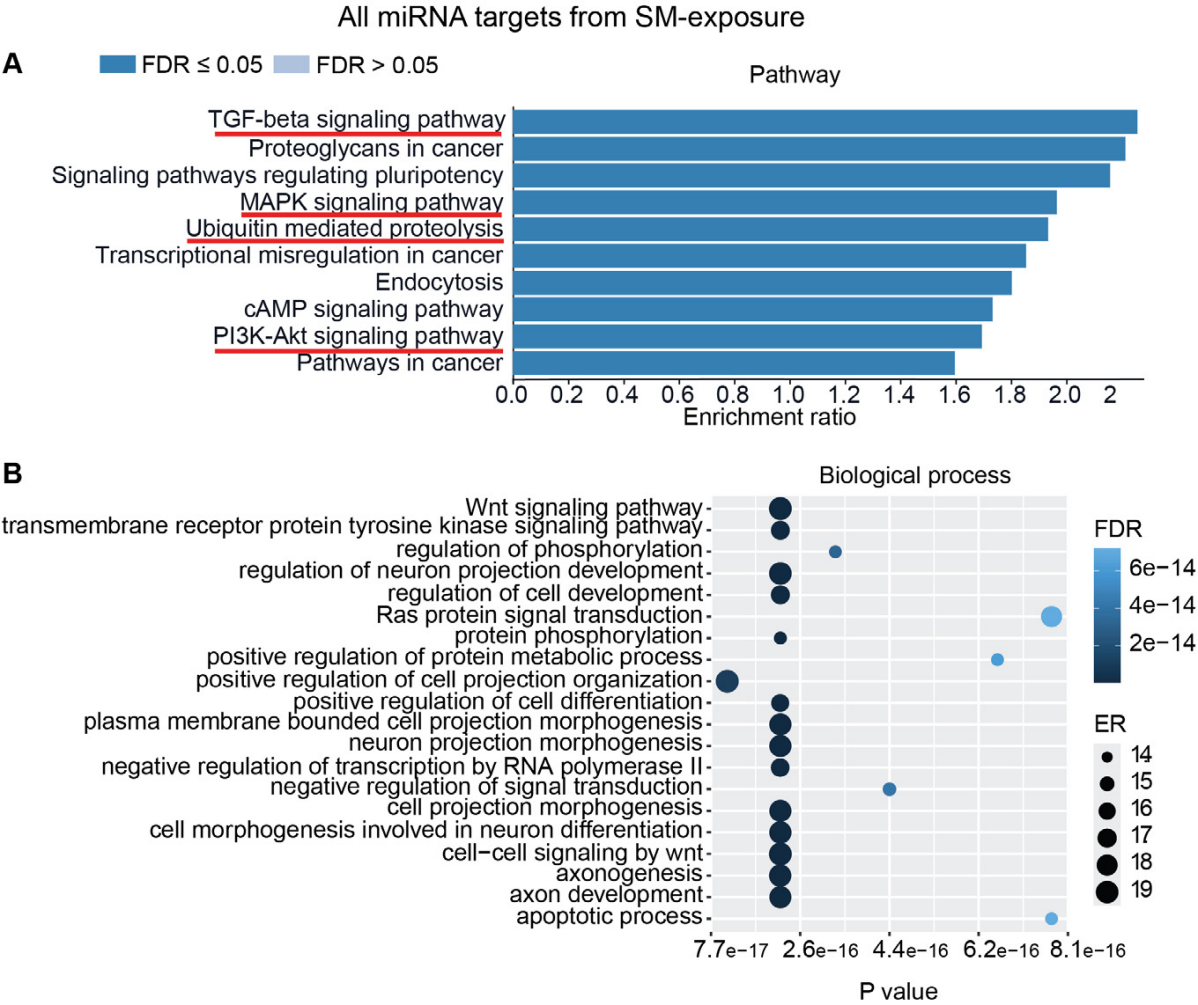
miRNAs target gene KEGG pathway enrichment analysis and GO analysis for SM exposure. miRNAs with RQ-fold ≥ 2 from all donors were subjected to target prediction (miRWalk), and common targets across all donors were identified and used to detect pathways and biological processes that were affected. Top 10 enriched pathways using the KEGG pathway from Webgestalt **(A)**, and GO analysis of common targets for 20 biological processes **(B)**. ER, enrichment ratio; FDR, false discovery rate. Underlined pathways in **(A)** were found modulated in all five donors when analyzing each donor separately. Graph in **(B)** was generated using ggplot2 in R.

To interpret the functional importance of the predicted miRNA target genes, an over-representation analysis of KEGG pathway and GO for the biological processes was performed using a web-based gene set analysis toolkit. The analysis of the pooled miRNA targets from five donors of ASC exposure revealed modulation of autophagy, MAPK signaling pathway (**Supplementary Figure S2**) (29), ubiquitin-mediated proteolysis, endocytosis, and PI3K/AKT signaling pathway (**Supplementary Figure S3**) among the top 10 weighted pathways (**Figure 5A**). These pathways were also found modulated when analyzing each donor separately. Additionally, other FDR significant pathways of relevance for TB were found to be involved, such as the HIF-1 signaling pathway, TNF signaling pathway, and mTOR signaling pathway. Similarly, in response to SM exposure, modulation of the TGF-beta signaling pathway (**Supplementary Figure S4**), MAPK signaling pathway, endocytosis, ubiquitin-mediated proteolysis, and PI3K/AKT signaling pathway were among the top 10 weighted pathways (**Figure 6A**), along with several other FDR significant pathways including the RAS signaling pathway, mTOR signaling pathway, and Wnt signaling pathway.

Furthermore, GO analysis for biological functions of the pooled miRNA targets from five donors in ASC exposure demonstrated that the affected biological processes were closely matching with that found by the KEGG pathway analysis (**Figures 5A, B**). Conversely, the GO analysis for biological functions of the pooled miRNA targets of five donors in SM exposure demonstrated more distinct biological functions among the top 20 significant biological processes that did not intuitively cover the MAPK signaling pathway or the PI3K/AKT signaling pathway found targeted by the KEGG pathway enrichment analysis of the same data (**Figures 6A, B**). The biological processes found affected by ASC and SM exposure showed a strong variation across helminth species to induce a miRNA-dependent response. Collectively, these results demonstrate that miRNAs in EVs from helminth antigen-exposed hMDMs modulate inflammatory pathways.

**Figure 7:**
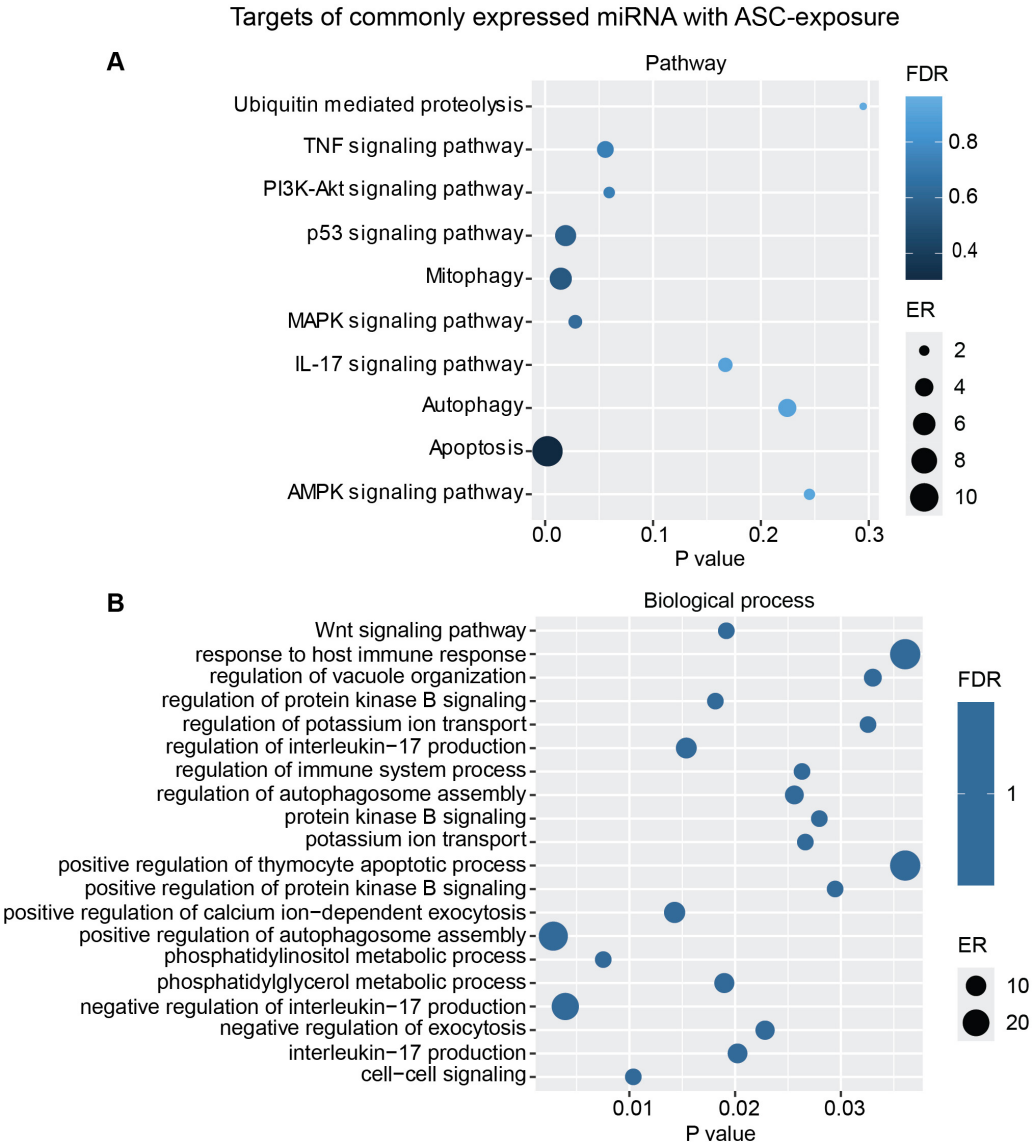
miRNAs target gene KEGG pathway enrichment analysis and GO analysis for the commonly expressed miRNA with ASC exposure. Commonly expressed miRNAs from all donors were subjected to target prediction (miRWalk), and common targets across all donors were identified and used to detect pathways that were affected using the KEGG pathway from Webgestalt **(A)**, and GO analysis of common target genes **(B)**. Graphs were generated using ggplot2 in R.

**Figure 8:**
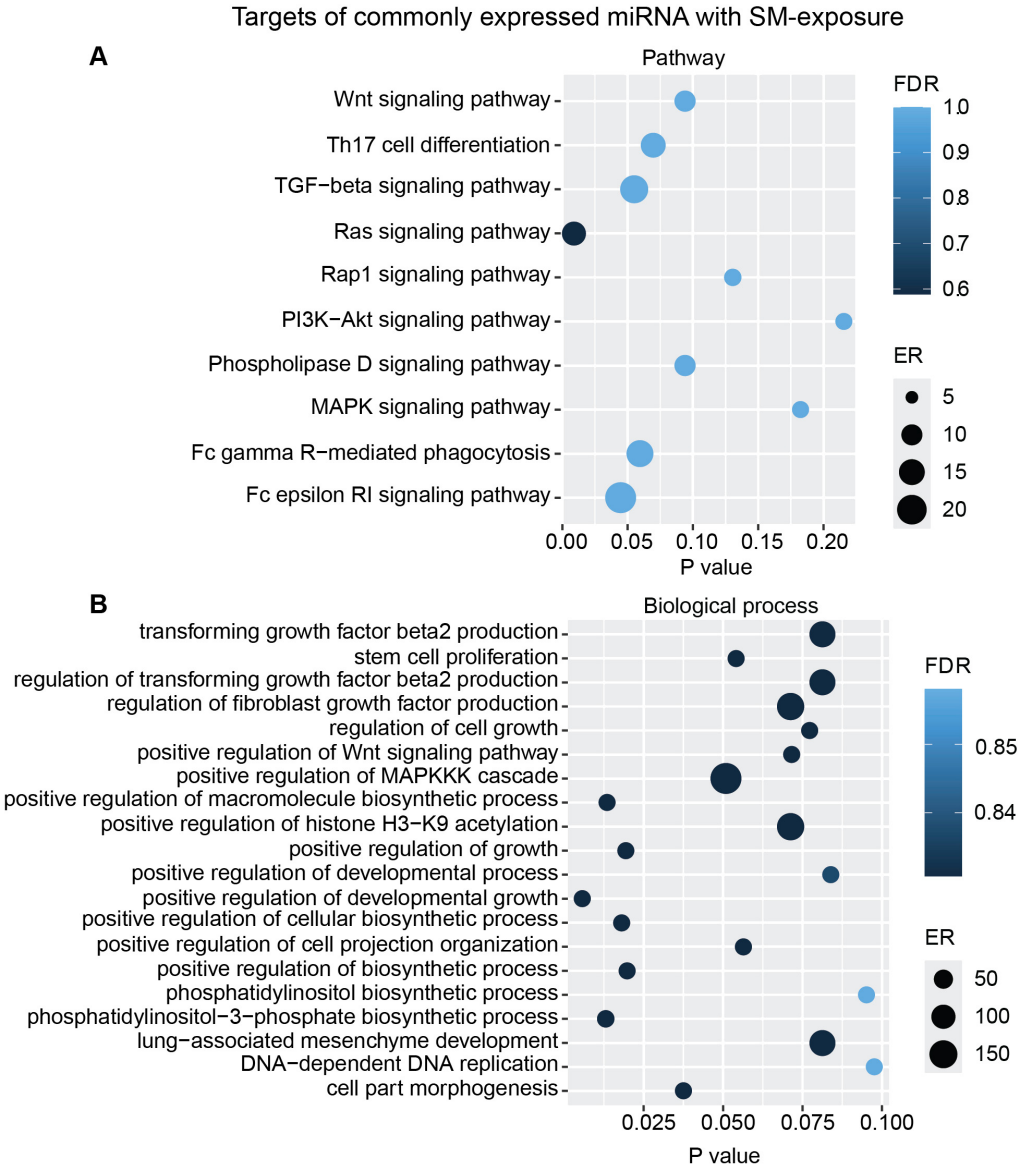
miRNAs target gene KEGG pathway enrichment analysis and GO analysis for the commonly expressed miRNA with SM exposure. Commonly expressed miRNAs from all donors were subjected to target prediction (miRWalk), and common targets across all donors were identified and used to detect pathways that were affected using the KEGG pathway from Webgestalt **(A)**, and GO analysis of common target genes **(B)**. Graphs were generated using ggplot2 in R.

Additional analysis using only the targets from the commonly overexpressed miRNA across all donors showed similar biological processes and pathways modulated with ASC exposure (**Figure 7**) as for SM exposure (**Figure 8**), with regard to MAPK signaling pathway and PI3K/AKT signaling pathway for ASC exposure and TGF-beta signaling pathway for SM exposure, respectively.

### 3.5 Key mRNA found targeted by commonly expressed miRNA in all donors

**Figure 9:**
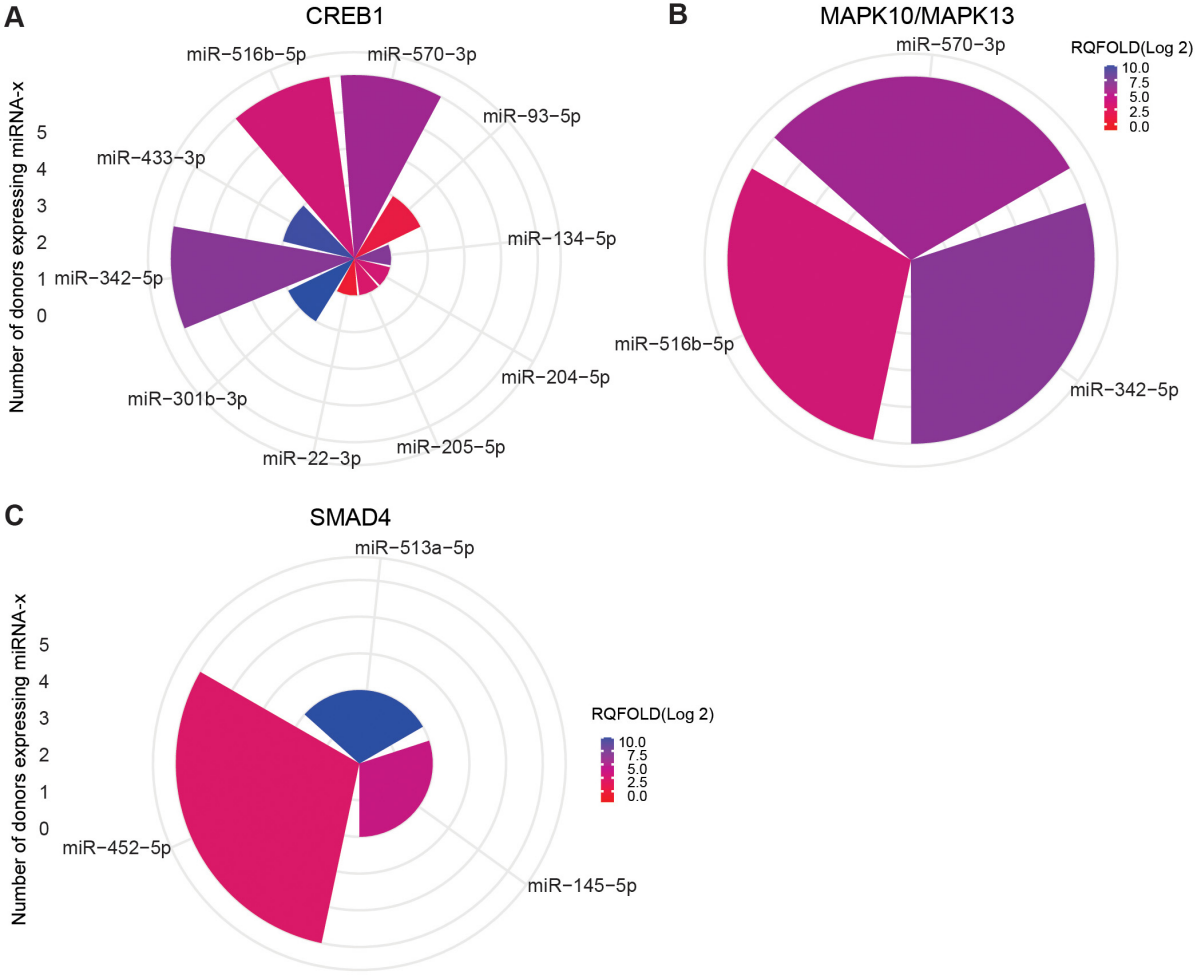
miRNA targeting CREB1 and MAPK10/13 in ASC exposure and SAMD4 in SM exposure of hMDMs. Commonly expressed miRNAs along with other miRNAs that target the same mRNA in the *n* = 5 donors analyzed. Target prediction using miRWalk showed CREB family **(A)** and MAP10/MAPK13 **(B)** targeted by miRNA upon ASC exposure and SMAD4 upon SM exposure **(C)**. Graphs were generated using ggplot2 in R.

To focus on mRNAs relevant to TB, we further screened for important mRNA targets across donors and identified CREB1, MAPK10, MAPK13, and SMAD4 as significant targets. CREB1, a crucial component of the PI3K/AKT pathway and a key transcription factor within the CREB family, was found targeted during ASC exposure. Ten miRNAs were found targeting CREB family, and 3 (miR-342-5p, miR-516b-5p, and miR-570-3p) out of 10 miRNA were found across all donors (**Figure 9A**). MAPK10 and MAPK13, also known as JNK3 and p38δ, respectively, belonging to the mitogen-activated protein kinase (MAPK) family and having a dominant role in cytokine production, phagocytosis, and antimicrobial response (23, 30), were also found targeted during ASC exposure. The same three miRNAs that targeted CREB were additionally found to target MAPK10 and MAPK13 (miR-342-5p, miR-516b-5p, and miR-570-3p) (**Figure 9B**). Of note, among the differentially expressed miRNAs from all conditions, these three miRNAs were the only ones found to target MAPK10 and MAPK13. During SM exposure, there was induction of three miRNAs that target SMAD4, out of which one miRNA (miR-452-5p) was overexpressed in all donors (**Figure 9C**). SMAD4 was found to be involved in regulating the TGF-beta signaling pathway.

## 4 Discussion

Helminth coinfection with TB has been recognized to influence the immune response against Mtb. However, the precise impact of helminths or their antigens on host-derived EVs and miRNAs remains unknown. These EVs and miRNAs could potentially modulate the immune system and macrophage control of Mtb growth. In our previous work, we discovered that pretreatment with *A. lumbricoides* antigens significantly altered Mtb growth control and macrophage inflammatory capacity. Specifically, conditioned medium derived from helminth-exposed macrophages demonstrated improved control over Mtb growth, accompanied by a reduction of infection-driven proinflammatory cytokines. Our major finding in the present investigation is that *A. lumbricoides* antigen exposure stimulates macrophages to release EVs and that these EVs were essential for enhancing macrophages’ intracellular Mtb growth control. Although EVs, or rather CD9-, CD63-, CD81-, and flotillin-1-expressing EVs, were released from macrophages to the cell culture medium regardless of stimulation, only EVs from Ascaris exposure significantly improved Mtb growth control and reduced infection-induced IL-1β release in treatment-naïve hMDMs. Across all donors tested, EVs from Ascaris-exposed macrophages overexpressed miR-342-5p, miR-516b-5p, miR-570-3p, and miR-188-3p. In contrast, EVs from *S. mansoni* antigen exposure resulted in the overexpression of miR-296-5p and miR-452-5p. These miRNAs play a crucial role in modulating intracellular signaling cascades involved in Mtb growth control and inflammation, notably targeting MAPK, PI3K/AKT, and TGF-beta signaling pathways. Overexpressed miRNAs were found to target crucial mRNA molecules, including CREB1, MAPK10, MAPK13, and SMAD4. Understanding these interactions between helminth-induced EVs, miRNAs, and macrophage responses may inform novel therapeutic strategies for TB management.

miRNA has been recognized for its modulatory role in host immune responses, and in the response against Mtb particularly, processes such as autophagy and apoptosis are targeted. For instance, miR-155 has been shown to inhibit apoptosis in cells of TB patients (15) and to contribute to autophagy-mediated clearance of mycobacteria by targeting Rheb (31). Although the six commonly expressed miRNAs found herein, to our knowledge, have not been studied in TB, they have been linked to the same pathways we found through target prediction analysis. For example, miR-342-5p was found to suppress PI3K/AKT in classically active macrophages and act anti-inflammatory in an atherosclerosis plaque mouse model (32), and in an acute kidney injury model, exosomes loaded with miR-342-5p alleviated inflammation by targeting TLR9 to promote autophagy (33). Additionally, miRNA-296-5p, which was overexpressed in EVs after *Schistosoma* antigen exposure, has been suggested to induce inflammation by activating NF-kB (34). Similarly, the other miRNAs found expressed by helminth antigen exposure have been seen to modulate the predicted pathways, e.g., autophagy (35), MAPK (36), and TGF-beta pathway (37).

Our target analysis based on the miRWalk data base revealed important targets affected by our miRNAs. CREB1, or cAMP Response Element-Binding protein 1, is a significant target within the PI3K/AKT pathway (**Supplementary Figure S3**) that regulates the expression of immediate early genes and blocks the nuclear localization of NF-kB p65, a crucial transcription factor involved in immune responses against Mtb and inflammation. CREB1 is rapidly activated in hMDMs upon Mtb infection, generating a favorable environment conducive to Mtb growth through the blockade of phagolysosomal fusion and inhibiting the necroptotic pathway. Furthermore, inhibiting CREB1 resulted in intact nuclear localization of NF-kB and enhanced macrophage Mtb growth control (38). In line with this, it was reported that the Mtb-induced CREB1 activation and modulation of inflammation through NF-kB p65 blockade in RAW murine macrophage-like cells was reversed by siRNA silencing of CREB, leading to enhanced Mtb growth control (39). Based on these findings and the fact that CREB1 was a common target for three of the miRNAs expressed across all donors after stimulation with ASC, this is an important mechanism for the enhanced Mtb growth control that we observe with EV-stimulation of Mtb infected hMDMs. The CREB finding is further supported by the biological function of the targeted genes as the PI3K/AKT signaling pathway was found modulated in all donors with Ascaris exposure.

Other important targets modulated by miRNAs that were overexpressed in EVs of Ascaris-exposed hMDMs was MAPK10 (JNK3) and MAPK13 (p38δ) (**Supplementary Figure S2**). These targets belong to the family of MAPK that are major players during inflammatory responses, especially in macrophages. p38δ MAPK was identified as a novel regulator of NLRP3 inflammasome activation in primary human macrophages that mediates IL-1β cleavage and secretion (40). Additionally, IL-1β secretion was decreased in response to LPS in bone marrow-derived macrophages from p38δ- MAPK-deficient mice (41). Moreover, it has been shown that p38δ inhibition or deletion leads to a blockade of CREB, that p38δ is essential for mitogen- and stress-activated kinase 1 (MSK1) phosphorylation or activation in bone marrow-derived macrophages, and that p38δ regulated MSK1 downstream targets that can limit inflammatory pathways downstream of TLRs (42). Thus, the observed decrease in Mtb infection-driven IL-1β release from hMDMs treated with EVs from Ascaris-antigen exposure aligns with the overexpressed miRNAs found targeting MAPK13 (p38δ). While IL-1β is generally associated with the restriction of intracellular Mtb growth (43) and IL-1β-deficient mice have higher bacterial loads when infected with Mtb (44), IL-1β signaling must operate within a narrow range as both excessive and defective IL-1β responses lead to lethal disease (45). EVs from Ascaris-exposed hMDMs thereby seem to lower the infection-driven IL-1β to an optimal range suitable for Mtb growth control. In fact, excessive IL-1β production and hyper-inflammation caused by various genetic polymorphisms have been associated with increased TB susceptibility, more severe forms of TB, including extrapulmonary TB, and a worsened treatment outcome (46, 47).

*Schistosoma*-induced miRNAs (miR-452-5p) were found to modulate the TGF-beta signaling pathway through the target SMAD4, which was common across all donors. TGF-β signaling is known to have an important function in macrophage polarization, immune regulation, and tissue homeostasis. SMAD-dependent TGF-β signaling pathways regulate M2 polarization, whereas SMAD-independent TGF-β signaling pathways regulate M1 polarization. SMAD4 is unexplored in the context of TB, but it was reported that SMAD4-dependant TGF-β signaling supressed TLR signaling, thereby interrupting pathogen recognition and induction of inflammatory responses (48). It was also previously reported that inhibition of SMAD4 resulted in significantly enhanced renal inflammation (49). Our miRNA targeting SMAD4 induced by *S. mansoni* antigen exposure is therefore indicative of an interrupted SMAD-dependant TGF-β signaling that could promote an inflammatory environment. In line with this, we previously showed that *S. mansoni* antigen exposure increased the M1 marker CCR7 on hMDMs with decreased IL-10 secretion when infected with Mtb (11). However, the effect of a manipulated TGF-β signaling (incurred by SMAD4 inhibition) during Mtb infection could be argued to have the strongest effect on immune responses during chronic TB infection when TGF-β is substantially elevated (50). TGF-β1 is the strongest profibrotic cytokine discovered (51), and TB granulomas can bear signs of TGF -β-driven fibrosis (52), where TGF-β levels are significantly higher in post-TB patients with pulmonary fibrosis (53). Therefore, local miR-452-5p administration strategies that modulate SMAD-dependent TGF-β pathways could be promising for reducing lung fibrosis in chronic inflammatory diseases, including TB.

In conclusion, we found that helminth antigen exposure of hMDMs generates EVs containing miRNA that target important immune pathways involved in inflammation and Mtb growth control. These effects were found to be helminth species-specific. Further mechanistic studies are needed to evaluate these miRNAs or their targets as biomarkers or possible targets during inflammatory diseases.

## Data Availability

The original contributions presented in the study are included in the article/**Supplementary Material**. Further inquiries can be directed to the corresponding author.
