# Supplementary material for: Macrophage-derived extracellular vesicles from Ascaris lumbricoides antigen exposure enhance Mycobacterium tuberculosis growth control, reduce IL-1β, and contain miR-342-5p, miR-516b-5p, and miR-570-3p that regulate PI3K/AKT and MAPK signaling pathways
Source: Front Immunol. 2024 Nov 6;15:1454881. doi: 10.3389/fimmu.2024.1454881 (PMC11576181; doi:10.3389/fimmu.2024.1454881)
Supplement: Supplementary file 1 [file DataSheet1.pdf]

Supplementary information

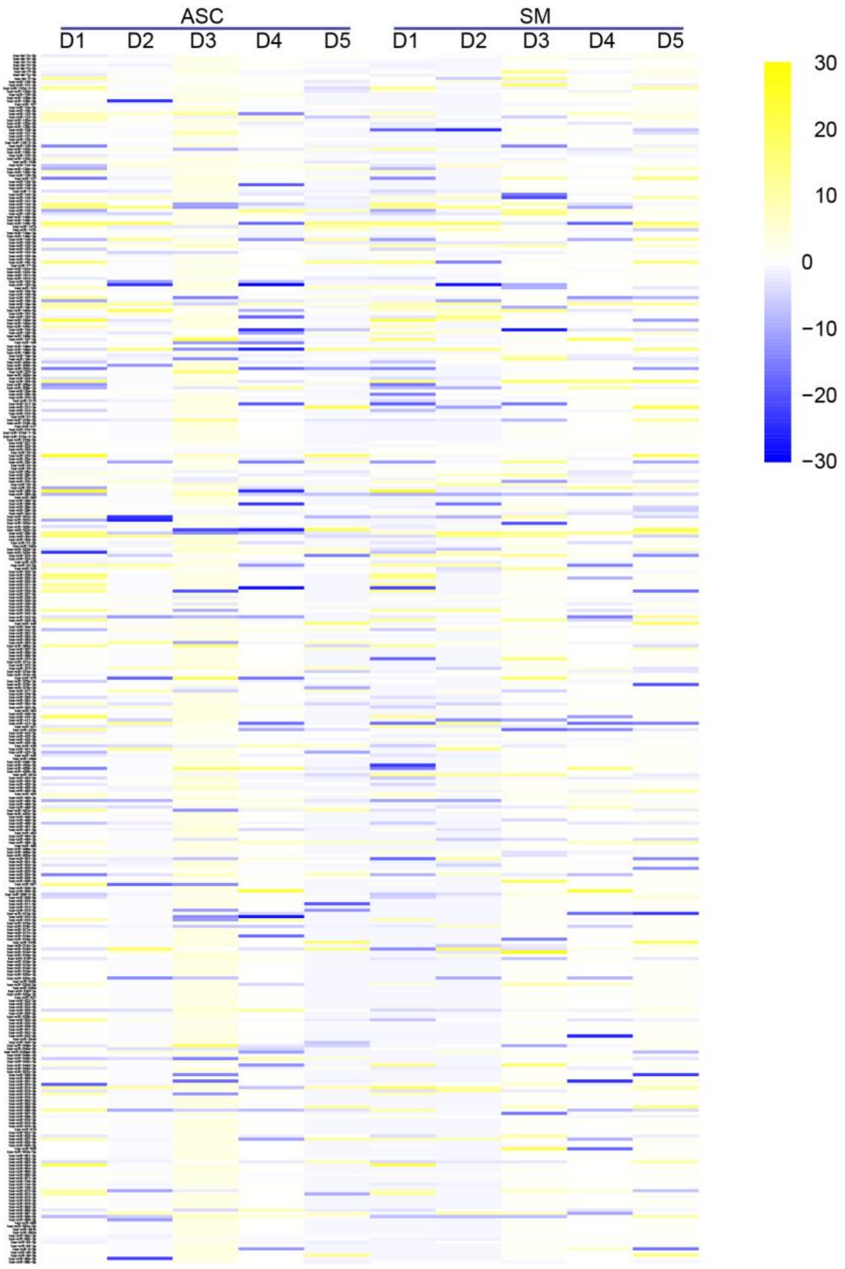

Supplementary Figure S1. The heat map of differentially expressed miRNAs from helminth antigen exposed macrophage derived EVs. The raw ΔΔCt values of the 377 miRNAs were used to generate the heat map without row/column normalization. Displayed color range, blue indicates downregulation, red indicates upregulation, and white represents expression levels similar to the control, n = 5. ASC, *Ascaris lumbricoides* antigen exposed hMDMs; D1-D5, hMDM-donor 1-5; and SM, *Schistosoma mansoni* soluble egg antigen antigen exposed hMDMs.

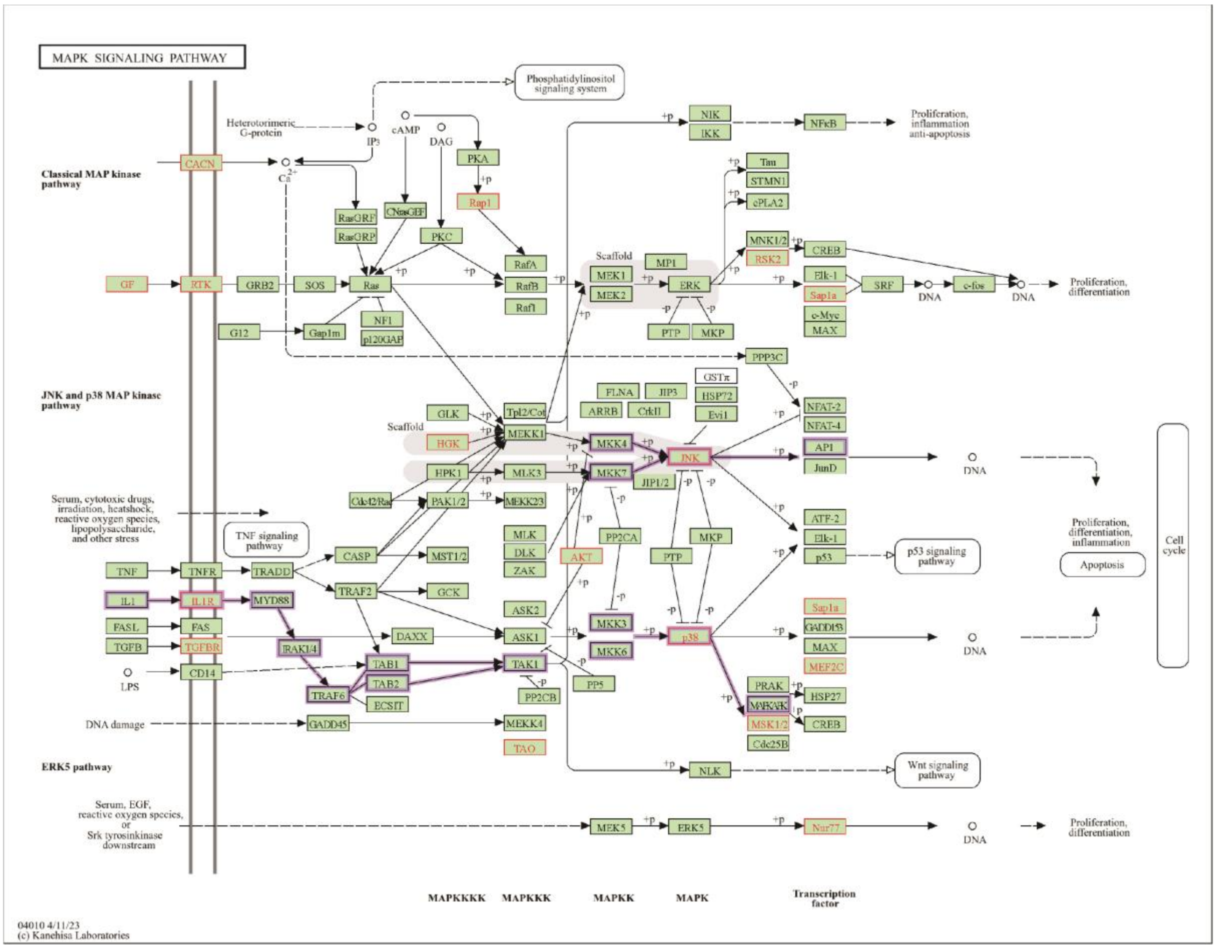

Supplementary figure S2. MAPK pathway regulated by miRNA targets from ASC-exposed hMDMs via MAPK10/MAPK13. Geographical diagram from KEGG pathway database for MAPK shows that IL-1-IL1R-p38 and IL-1-IL1R-JNK signaling pathway are regulated (lines colored blue). Targeted genes are shown in red color.

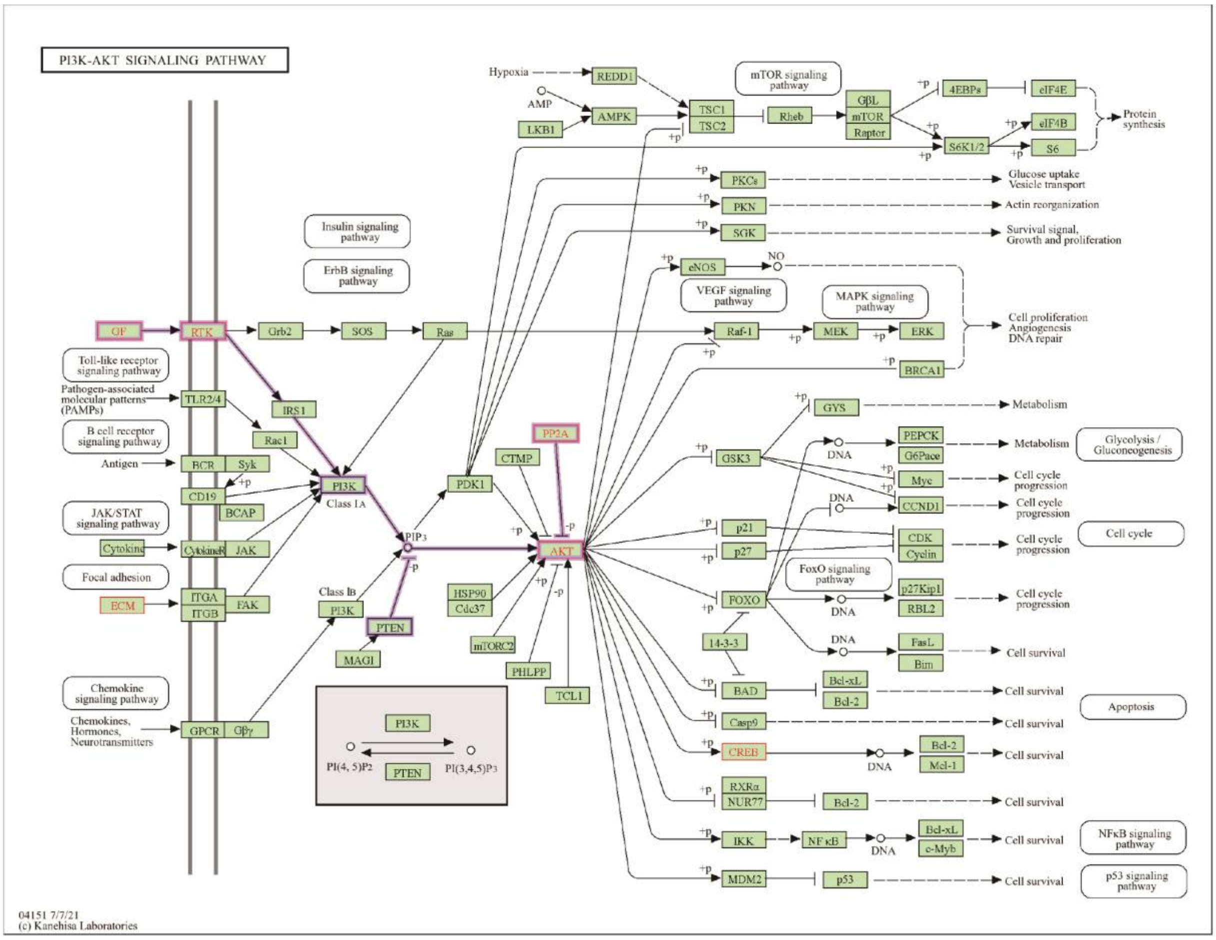

Supplementary figure S3. PI3K/AKT pathway regulated by miRNA targets from ASC-exposed hMDMs via CREB1. Geographical diagram from KEGG pathway database for PI3K/AKT signaling pathway. GF-RTK-PI3K signaling pathway, and PP2A-AKT signaling pathway are regulated (lines colored blue). Targeted genes are shown in red color.

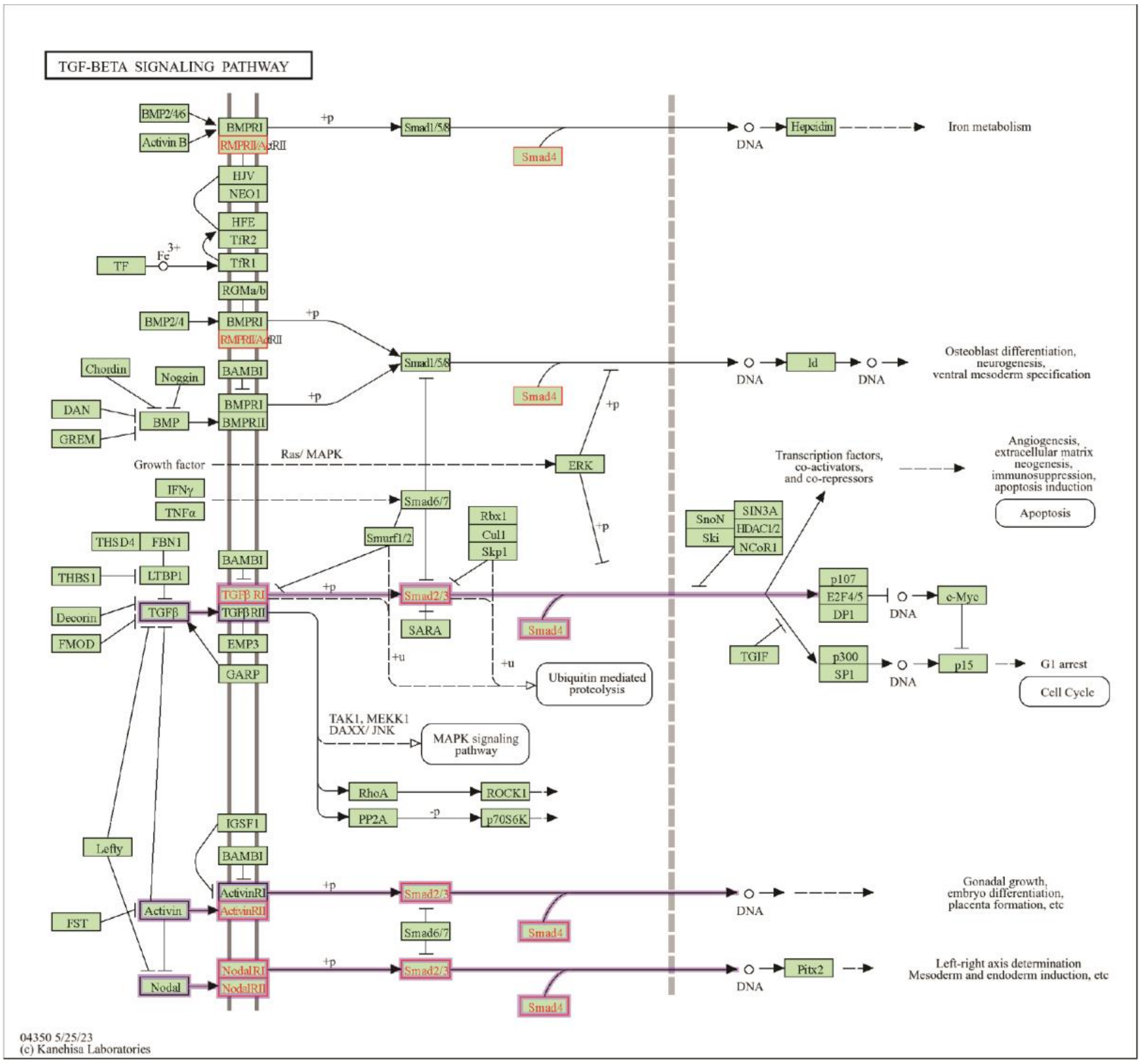

Supplementary figure S4. TGF-beta signaling pathway regulated by miRNA targets from SM-exposed hMDMs via SMAD4. Geographical diagram from KEGG pathway database showing TGF-b signaling pathway. TGF-b signaling pathway, Activin signaling pathway and Nodal signaling pathway are regulated (lines colored blue). Targeted genes are shown in red color.

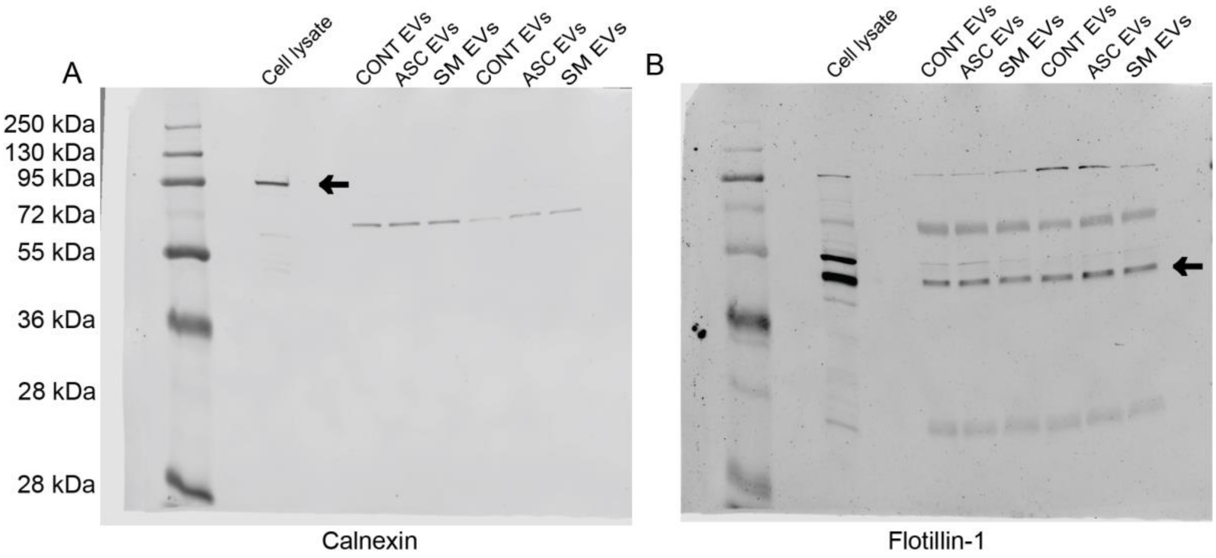

Supplementary figure S5. Isolated EVs carry flotillin-1 but not the impurity marker calnexin. Isolated EVs were lysed in sample buffer and subjected to western blot using anti-flotillin-1(EV marker) and anti-calnexin (endoplasmic reticulum protein). Full scan of the entire original gel(s) that were shown cropped in Figure 3B, for calnexin **(A)**, and flotillin-1 **(B)**. Arrows added in **(A)** and **(B)** indicate actual protein according to its molecular weight. For both **(A)** and **(B)**: lane 1 = molecular standard, lane 2 = empty, lane 3 = cell lysate, lane 4 = empty, lane 5 = EVs released from healthy human monocyte-derived macrophage (hMDMs) that are unexposed (CONT EVs), lane 6 = EVs from *Ascaris lumbricoides* antigen exposed hMDMs (ASC EVs), lane 7 = EVs from *Schistosoma mansoni* antigen exposed (SM EVs). Lane 3-7 shown in Figure 3B.
